# Comparative Study of Limberg Flap Reconstruction With Wide-Open Excision and Healing by Secondary Intention in the Management of Pilonidal Sinus: Our Experience at a Tertiary Care Center in India

**DOI:** 10.7759/cureus.26396

**Published:** 2022-06-28

**Authors:** Swapnil P Chopade, Geet R Adhikari

**Affiliations:** 1 Department of General Surgery, Om Multispeciality Hospital, Malkapur, IND

**Keywords:** wide excision, sacrococcygeal sinus, secondary intention healing, pilonidal, primary closure, limberg flap

## Abstract

Introduction

A pilonidal sinus (PNS) is a small passageway in the subcutaneous tissue which develops most frequently in the sacrococcygeal area. In terms of postoperative outcomes, the decision on the best surgical treatment for PNS is still a challenge for a surgeon. Prevention of the disease recurrence and improving quality of the life can be considered primary goals of the treatment. The current study intends to compare two commonly practiced surgical treatments for PNSes-Rhomboid excision with Limberg flap repair against wide-open excision with healing by secondary intention.

Methods

In a prospective randomized study, 50 patients with sacrococcygeal PNS were divided into two groups. Group A was operated by rhomboid excision with Limberg flap reconstruction and Group B was operated by wide-open excision and healing by secondary intention. Data were collected on a specially designed structured proforma and consisted of patient demographics, medical history, presentation, and postoperative complications assessed for a period of 6 months. Comparative outcomes of interest were postoperative pain, postoperative anxiety, duration of wound healing, duration of work loss, presence of wound infection, and recurrence.

Results

Mean age of 28 years was observed across the study with a male preponderance (76%). The mean visual analog scale (VAS) score for pain was greater in Group A during the early postoperative period, i.e., days 1, 3, and 7. However, patients in Group B reported a mean VAS score of 3 ± 0 and 1 ± 0 at one month and 2 months, respectively indicating a longer duration of postoperative pain overall. Patients in Group B also reported a significantly higher VAS for anxiety (VAS-A) score for postoperative anxiety/stress in all the follow-up visits. The mean healing time was 20 ± 2 days in Group A and 57 ± 11 days in Group B showing a significant difference. Duration of work loss was also significantly higher in Group B (31 days). Five patients in Group B developed wound infections. No recurrence was observed across both the groups in this study.

Conclusion

According to the findings of this study, the Limberg flap method outperforms the wide-open excision approach in terms of healing duration, work loss days, postoperative pain, anxiety, and wound infection. Both the techniques, however, are comparable in terms of recurrence.

## Introduction

Pilonidal refers to a “nest of hairs” while sinus refers to a tract. Herbert Mayo originally defined sacrococcygeal pilonidal disorders as a hair-containing sinus in 1833. Because the condition was so frequent among jeep drivers during World War II, it was dubbed jeep disease [1]. A pilonidal sinus (PNS) is a small passageway beneath the skin that is commonly present in the sacrococcygeal area. Damaged hair follicles and imprisoned hair are thought to be the cause, leading to folliculitis, infection, and rupture into the surrounding subcutaneous tissue. PNS is a painful, persistent condition that is prone to recurrence [2]. The pilonidal disease was once assumed to be a congenital ailment produced by aberrant skin in the gluteal cleft, but it is now thought to be an acquired syndrome caused by hair in the cleft [3]. The hair triggers an inflammatory response, resulting in persistent sinus and tract drainage, as well as secondary infection and abscess formation [4]. It is now largely accepted that the condition is acquired and caused by local trauma, poor hygiene, extreme hairiness, or the existence of a deep natal cleft [5]. Males, young age group, overweight, and hirsute individuals, as well as those with a positive family history, are more likely to acquire this benign condition [6]. Due to differences in hair features and growth patterns, the disease is more common in Caucasians than in Asians or Africans [7]. PNS, according to some experts, could be caused by a congenital pilonidal dimple. Pilonidal disease is characterized by visible pits in the midline of natal clefts, which resemble larger hair follicles under the microscope. The expansion is induced by the stretching of the follicular openings caused by gravity pulling on the buttocks. Sweating excessively might also lead to the development of PNS. Moisture can fill a stretched hair follicle, creating a low oxygen environment that encourages anaerobic bacteria development, which is common in the PNS. Bacteria and low oxygen levels impede wound healing and hasten the formation of the PNS [8].

PNS disease can be asymptomatic, acute, chronic, or recurrent. The common presenting symptoms are pain (84%), discharge (78%), and localized edema [9]. However, to rule out other differential diagnoses such as perirectal abscess, anal fistula, hidradenitis suppurativa, and pyoderma gangrenosum; a thorough clinical examination, history taking, and radiological investigations like MRI may be necessary. PNS is linked to a high rate of morbidity and has a major socio-economic impact on those who are affected [10]. Various surgical techniques for sacrococcygeal PNS disease have been tried, including laying the track open, chemical treatment with phenol, marsupialization, wide excision, excision with primary simple midline or asymmetric closure, and techniques involving various plastic procedures. The best treatment for this disease is debatable because all of the existing options have their share of advantages and disadvantages. So yet, no globally accepted and perfect treatment modality has been established, and in the majority of cases, the surgeon relies on his own surgical experience. The ideal treatment method should take less time, cost less money, have fewer postoperative problems, short hospital stay, quick return to work, and have the least probability of recurrence [11]. Every procedure is associated with a set of complications. Recurrence is the main complication; others are infection with wound dehiscence and postoperative pain. The rate of recurrence varies greatly depending on the clinical presentation, whether acute or chronic, and the surgical treatment modality [12]. The Limberg flap is a transposition flap for covering a rhomboid-shaped defect with opposite sides of equal length, opposite medial and lateral angles of 120°, and superior and inferior angles of 60°, as developed by Alexander Limberg in 1948. This enables a tension-free closure of the rhomboid-shaped defect and donor site [13,14]. According to several studies, this is the best treatment for chronic sacrococcygeal PNS disease in terms of disease recurrence, flap failure, and postoperative complications [15].

Less invasive endoscopic treatments have been developed for a better-targeted treatment of the condition. The goal of such treatments is to lessen the morbidity associated with excisional surgery while demonstrating success in eradicating disease-causing variables. Meinero et al. described endoscopic pilonidal sinus therapy (EPSiT) in 2014, along with Video-Assisted Ablation of Pilonidal Sinus (VAAPS), which provided a magnified view of the tracts (Meinero P, Mori L, Gasloli G: “Endoscopic pilonidal sinus treatment (E.P.Si.T).” Tech Coloproctol, 2014, 18: 389-92). The procedure is divided into two phases: diagnostic and interventional. The goal of EPSiT/VAAPS is to target the hair invagination that drives PNS disease pathogenesis and inhibits further growth in the tract, which cures by secondary intention. This new technology still faces challenges, such as standardizing techniques to reduce heterogeneity and getting high-quality prospective trial data. Furthermore, the costs of establishing an endoscopic technique and the learning curve for surgeons take longer to accomplish, as well as the possibility of repeat treatments must be considered [16].

This study aims to compare the postoperative outcomes of the Limberg flap reconstruction technique with wide-open excision and healing by secondary intention in the surgical management of PNS.

## Materials and methods

This study was carried out on 50 patients with PNS who visited the Department of Surgery, Om Multispeciality Hospital, Malkapur, Maharashtra within the study time period of one year. The institutional ethics committee (Om Hospital and Research Center Ethics Committee) issued the approval for this study on 2/2/2021 with an approval number ECR/694/INST/MH2021-RR23 for a period of one year. All the cases were thoroughly examined and investigated to be considered appropriate to be a part of this study. The patients were divided into two groups of 25 each (Groups A and B). Patients in Group A underwent Limberg flap reconstruction surgery, while patients in Group B underwent wide-open excision surgery with healing by secondary intention. The nature of surgical procedures was explained to the patients and informed consent was taken. Patients with recurrent disease, previously operated patients and immunocompromised patients, or patients with any other significant comorbidities like diabetes mellitus which may have altered the interpretations were excluded from the study.

Preoperative care, anesthetic evaluation, and postoperative follow-up were performed on all patients under the same clinical guidelines by the same surgeon. The following documents were reviewed: outpatient clinical notes, discharge summary, operation notes, and laboratory results. All patients were discharged on Day 3 postoperatively and were followed up for 6 months to assess results and generate a comparison. Data were collected and entered simultaneously in the statistical package for social sciences (SPSS; IBM, Armonk, New York, USA) version 23 and coded appropriately. Descriptive statistics were calculated to summarize the sample characteristics in terms of frequency and percentage. Analytical and inferential analysis was applied between a dependent variable and other independent variables. Significance was set at standard 0.05.

Group A intervention: Limberg flap reconstruction surgery

**Figure 1:**
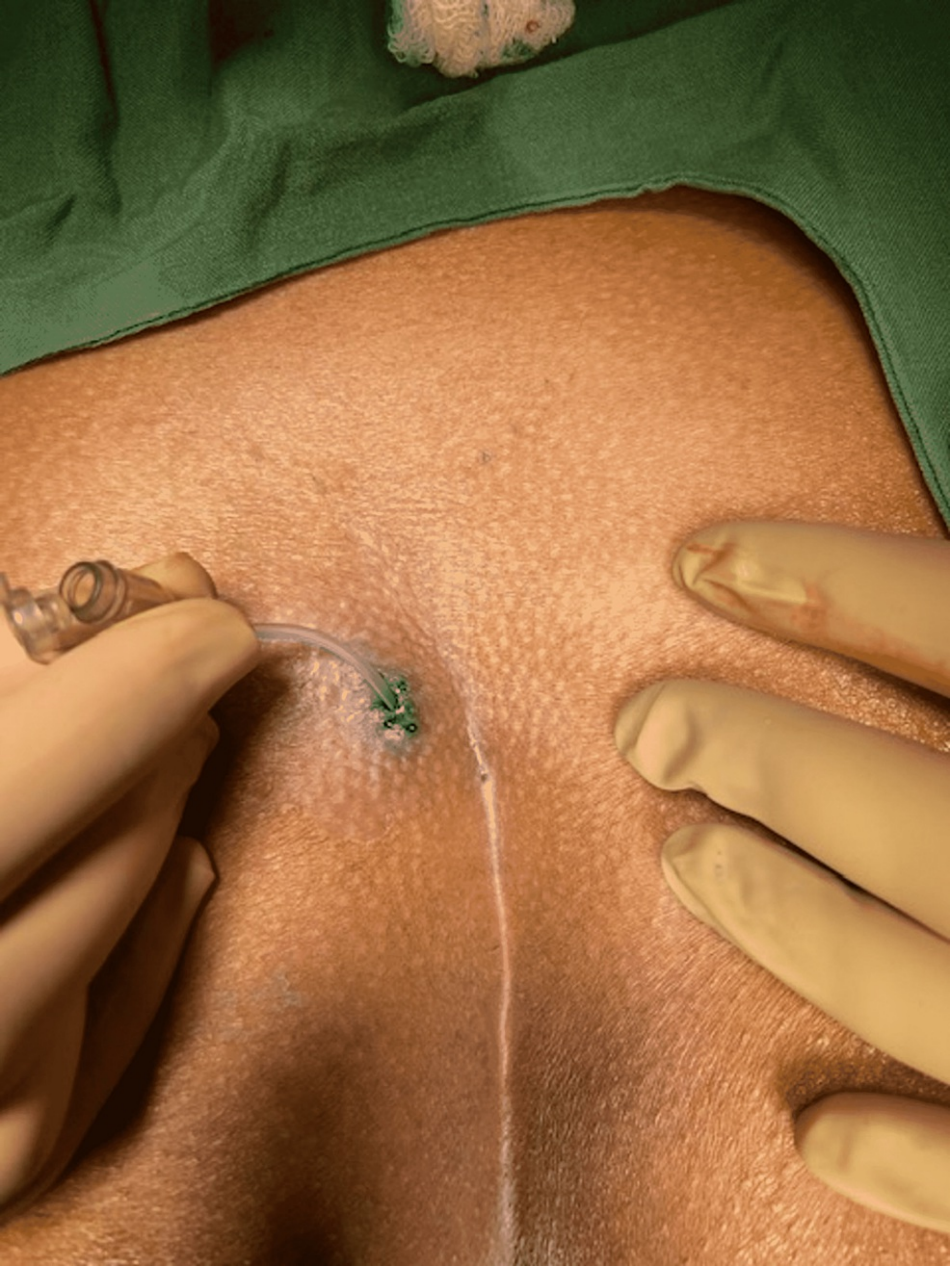
Methylene blue dye being injected into the pilonidal sinus opening

Methylene blue was injected into the external opening of the sinus and thoroughly massaged to achieve uniform diffusion of the color (Figure 1).

**Figure 2:**
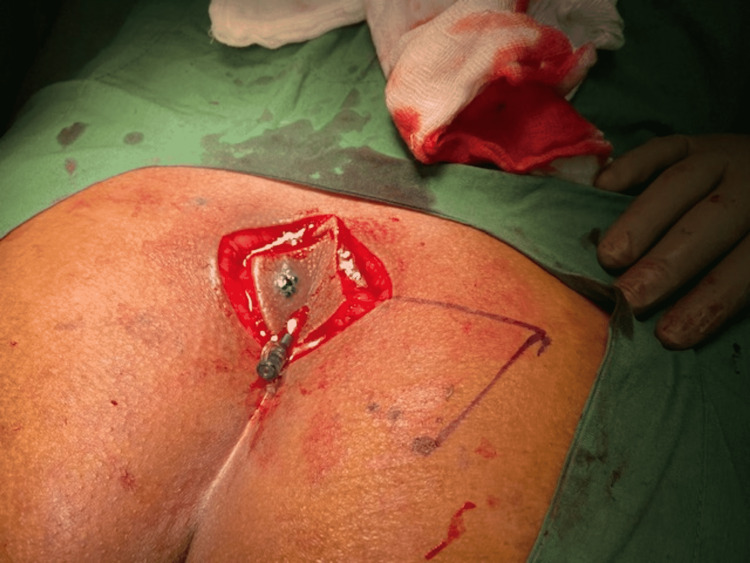
Limberg flap marking done after developing rhomboid excision

The skin was marked for a rhomboid-shaped excision and the entire sinus tract was removed deep down to the presacral fascia. Incorporating gluteal fascia, a right- or left-sided Limberg transplantation flap was fully mobilized on its inferior border and delivered medially to cover the rhomboid defect (excised area) (Figure 2).

**Figure 3:**
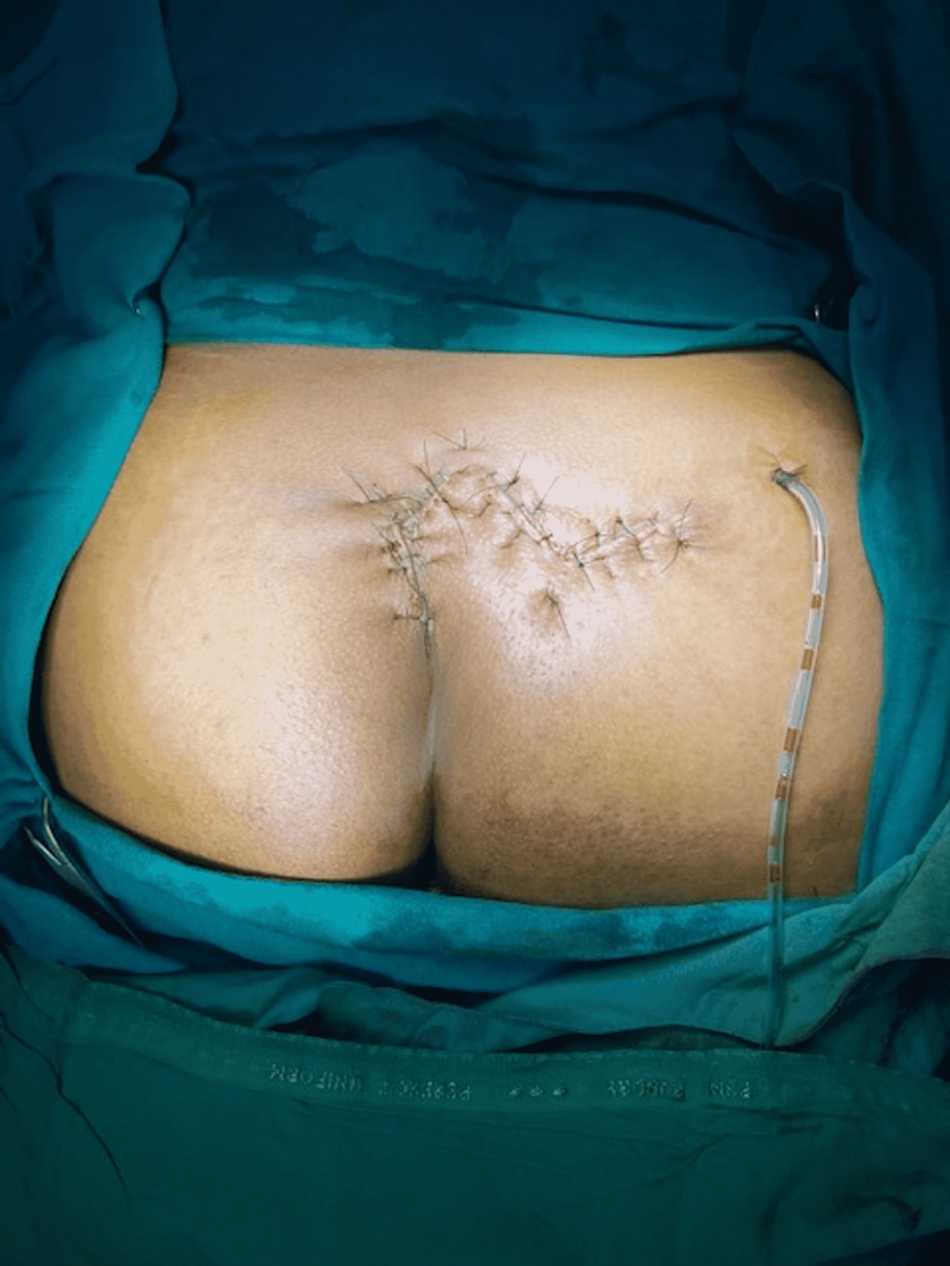
Limberg flap closure done with negative drain fixation

A negative suction drain (size 16) was kept in the wound cavity and fixed to the skin with a silk suture in all the cases. Polyglactin and nylon interrupted sutures were used to approximate subcutaneous tissue and skin, respectively, without tension (Figure 3). The drain was removed on the seventh day. Skin sutures were removed after a delayed period of 3 weeks postoperatively in all the patients.

Group B intervention: Wide-open excision with healing by secondary intention

Following the injection of methylene blue into the sinus tract, wide excision of an elliptical wedge of skin and subcutaneous tissue was made and developed down to the presacral fascia to remove all inflammatory tissue, as well as the sinus tract and debris, allowing the wound to granulate from the base. The wound cavity was not closed but rather left open to heal by secondary intention. The patients in this group were advised and explained about the need for daily dressing of the wound at home post-discharge. A relative of the patient’s preference was chosen, assigned, and trained to assist with the dressing to maintain uniformity in the daily dressing process and minimize observation bias later during wound assessment.

The time taken for wound healing was measured from the time of incision to the time of wound closure (Group A) or epithelization (Group B). Pressure dressings, a reduced residue diet till postoperative Day 3, and dressing examination on postoperative Days 3 and 7 were all part of the postoperative treatment. Discharge instructions included avoiding extended sitting, riding bicycles or scooters until 6 weeks postoperatively to prevent wound disruption, increasing local cleanliness, and shaving or using depilatory cream daily.

## Results

In this study, most of the study participants were in the age group of 18-27 years in both groups. Male preponderance (76%) was observed in the study with the mean age of study participants of 28 years. The mean age in the Limberg flap group was 28±7 years and that in the wide excision group was 28±6 years. There was not much difference in the mean age of both the groups as participants were randomly distributed in each group. Comparison of visual analog scale (VAS) scores for pain among study participants of both the groups showed that on postoperative Days 1, 3, and 7 mean VAS score was higher for Group A as compared to Group B, depicting more pain among the study participants who underwent Limberg flap reconstruction surgery. Although, during follow-up visits at 1,2,3, and 4 months study participants in Group A did not complain of any pain. At 1 and 2 months follow-ups, the mean VAS score observed by study participants in Group B was 3 ± 0 and 1 ± 0, respectively (Table 1).

**Table 1:** Comparison of mean VAS scores for postoperative pain among different groups VAS: visual analog scale

VAS score	Group A	Group B
	Mean ± SD	Mean ± SD
Day 1	8 ± 1	7 ± 1
Day 3	8 ± 1	7 ± 1
Day 7	5 ± 1	4 ± 1
1 Month	0	3 ± 0
2 Months	0	1 ± 0
3 Months	0	0
4 Months	0	0

On comparing the VAS for anxiety (VAS-A) score for postoperative anxiety/stress, higher mean scores were observed in study participants of Group B who underwent wide excision, depicting higher anxiety/stress levels among them. Study participants in Group A also reported significant mean VAS-A scores till one week due to the presence of negative drain and sutures. The participants, however, reported no anxiety after drain and suture removal at 1, 2,3, and 4 months visit. A mean VAS-A score of 10 ± 14 at 4-month follow-up was still reported by study participants in Group B which could be attributed to the long time taken for wound healing, and the psychological stress of daily dressings in such patients (Table 2).

**Table 2:** Comparison of mean VAS-A scores for postoperative anxiety among different groups VAS-A: visual analog scale for anxiety

VAS-A score	Group A	Group B
	Mean ± SD	Mean ± SD
Day 1	83 ± 8	87 ± 5
Day 3	73 ± 8	81 ± 6
Day 7	59 ± 6	72 ± 6
1 Month	0	56 ± 7
2 Months	0	46 ± 6
3 Months	0	36 ± 11
4 Months	0	10 ± 14

In the present study, mean healing time in Group A was 20 ± 2 days and in Group B was 57 ± 11 days and this difference in mean was statistically significant. Also, days of work loss were significantly less in Group A, i.e., 22 ± 2 days as compared to Group B, i.e., 31 ± 5 days (Table 3).

**Table 3:** Comparison of mean of days of work loss and time taken for wound healing across different groups

	Group A	Group B	t	p-value
	Mean ± SD	Mean ± SD		
Days of work loss	22 ± 2	31 ± 5	7.606	0.000
Time for wound healing (days)	20 ± 2	57 ± 11	16.401	0.000

No cases of wound infection were seen in study participants who underwent Limberg flap reconstruction surgery. However, 20% of the study participants who underwent wide-open excision surgery with healing by secondary intention (Group B) had wound infection. None of the study participants in our study had recurrence of the disease.

## Discussion

So far, a wide range of procedures, from simple curettes to sophisticated flap techniques, have been documented for PNS. An ideal surgery, in addition to clearing the disease, should also remove the natal cleft, removing the anatomical inclination for the sinus to recur. However, none of the strategies has been proven to be superior to others in every way. PNS is most frequent after puberty in males, typically in the second and third decades of life [17]. In accordance with the literature, in the present study, a majority of the participants were in the age group of 18-27 years with a male preponderance. Many of them gave a history of long sitting duration at their respective jobs and had an increased hair growth in the sacrococcygeal region. Similar results were reported by Singh et al., where a majority of patients were in the age group of 20-30 years and most of the patients were males [17]. Sondenaa et al. obtained similar results when they evaluated 322 individuals with pilonidal disease prospectively and calculated that the incidence is 10 times higher in men than in women [18]. On Days 1, 3, and 7, the mean VAS score for pain in Group A was greater than in Group B, indicating that the study participants who received Limberg flap reconstruction surgery experienced more pain postoperatively. This can be explained by the presence of nonabsorbable nylon sutures and drain fixation over the skin causing more pain. The pain, however, significantly reduced after the removal of drain and sutures, hence the majority of the participants in Group A reported a VAS score of zero during follow-up visits at 1, 2, 3, and 4 months. In a study done by Alam et al., the authors reported that 66.7% and 33% of the study participants respectively reported pain in Group A (Limberg procedure) and Group B (wide excision) [19].

Also, in the present study, when comparing mean VAS-A scores for postoperative anxiety levels, study participants in Group B who underwent wide excision had higher mean scores, indicating more anxiety among them. This could be attributed to the presence of an open wound, the need for daily dressing in presence of a relative, and anticipation of wound healing as early as possible. In the present study, there were no cases of wound infection among study participants who had Limberg flap reconstruction surgery (Group A). However, despite regular antiseptic daily dressing, wound infection occurred in 20% of the study participants who received wide-open excision surgery. Jabbar et al. reported in their study that 20% of patients in open procedure and 16.67% of patients in Limberg flap procedure developed wound infections [20]. Similarly, Alam et al. also reported 33% infection in wide excision and only 13% in Limberg procedure [19]. Singh et al. reported five patients in the Limberg flap group and six patients in the broad excision group who suffered wound infections, thereby lengthening their hospitalization stay [17].

In the current study, the mean healing time in Group A was 20± 2 days and 57± 11 days in Group B, and this difference was statistically significant. Days of work lost were also significantly smaller in Group A (22 ±2 days) than in Group B (31± 5 days). The patients who received Limberg flap repair could return to their work after suture removal, however, patients with wide local excision resisted resuming their work due to significant anxiety about the wound and the requirement of daily dressing. In the study done by Kumar et al., the mean healing time for group II (rhomboid excision with Limberg flap reconstruction) was 17.0±8.0 days and for group III (open excision and healing by secondary intention) was 60±9.6 days which is in accordance with our findings [9]. In the present study, no recurrence was reported in either group. Similar results were reported by Kumar et al. where none of the study participants of either group reported recurrence [9]. On contrary, Singh et al. reported 16% recurrence in the wide excision group [17]. Alam et al. also reported 33% recurrence in the wide excision group and only one case of recurrence in the Limberg procedure [19].

A few limitations can be explained in context with the present study. The small sample size is an obvious limitation. The majority of patients were from the Malkapur region and adjacent areas; as a result of which the sample does not reflect the full Indian population. Also, the study solely compares two methods of PNS surgery; other operations were not included. The topic of cost-effectiveness is also not addressed in this study and will have to be reviewed individually.

## Conclusions

The Limberg flap method outperforms the wide excision approach in terms of wound healing duration, work loss days, postoperative anxiety, and the possibility of wound infection. These findings are most likely due to the advantage of the Limberg flap procedure which removes not only the primary sinus but also flattens the natal cleft thereby lowering the possibilities of hair build-up, mechanical discomfort, and recurrence. However, the Limberg flap approach may cause significant pain during the early postoperative period as per the findings of this study. To conclude, despite requiring difficult surgical skills, rhomboid excision with Limberg flap is a preferred treatment for PNS illness due to its low recurrence rates and few complications. Other benefits include speedy recovery, a short hospital stay, and an early return to daily life.
